# Novel Therapies of Hepatitis B and D

**DOI:** 10.3390/microorganisms9122607

**Published:** 2021-12-17

**Authors:** Iman Waheed Khan, Mati Ullah Dad Ullah, Mina Choudhry, Mukarram Jamat Ali, Muhammad Ashar Ali, Sam L. K. Lam, Pir Ahmad Shah, Satinder Pal Kaur, Daryl T. Y. Lau

**Affiliations:** 1 Liver Center, Department of Medicine, Beth Israel Deaconess Medical Center, Harvard Medical School, Boston, MA 02115, USA; ikhan1@bidmc.harvard.edu (I.W.K.); mullah@bidmc.harvard.edu (M.U.D.U.); mchoudhr@bidmc.harvard.edu (M.C.); mali7@bidmc.harvard.edu (M.J.A.); mali8@bidmc.harvard.edu (M.A.A.); skaur3@bidmc.harvard.edu (S.P.K.); 2 Liver Center, Department of Medicine, Department of Pharmacy, Beth Israel Deaconess Medical Center, Harvard Medical School, Boston, MA 02115, USA; sllam@bidmc.harvard.edu; 3 Department of Internal Medicine, University of Texas, San Antonio, TX 78229, USA; shahp2@uthscsa.edu

**Keywords:** Hepatitis B virus (HBV), Hepatitis D virus (HDV), novel therapies, siRNAs, cccDNA inhibitors, capsid assembly modulators, entry and exit inhibitors, interferon, NRTIs, immunomodulators

## Abstract

Hepatitis B virus (HBV) infection is a global public health issue and is a major cause of cirrhosis and hepatocellular carcinoma (HCC). Hepatitis D virus (HDV) requires the hepatitis B surface antigen (HBsAg) to replicate. The eradication of HBV, therefore, can also cure HDV. The current therapies for chronic hepatitis B and D are suboptimal and cannot definitely cure the viruses. In order to achieve functional or complete cure of these infections, novel therapeutic agents that target the various sites of the viral replicative cycle are necessary. Furthermore, novel immunomodulatory agents are also essential to achieve viral clearance. Many of these new promising compounds such as entry inhibitors, covalently closed circular DNA (cccDNA) inhibitors, small interfering RNAs (siRNAs), capsid assembly modulators and nucleic acid polymers are in various stages of clinical developments. In this review article, we provided a comprehensive overview of the structure and lifecycle of HBV, the limitations of the current therapies and a summary of the novel therapeutic agents for both HDV and HBV infection.

## 1. Introduction

Hepatitis B virus (HBV) is an enveloped partially double-stranded hepatotropic DNA virus belonging to the family of Hepadnaviridae. WHO has estimated that about 296 million people were chronically infected with HBV in 2019, and each year there is a surge of about 1.5 million new HBV infections around the world despite it being a vaccine-preventable disease [1]. Acute hepatitis B in adults is usually self-limiting with less than 5% developing chronic hepatitis, but it can lead to fulminant liver failure in about 1–2% of acute infections [2]. In contrast, approximately 90% of the acute hepatitis B acquired in the first year of life developed chronic hepatitis [3]. Chronic hepatitis B can progress to cirrhosis, liver failure and hepatocellular carcinoma. In 2019, complications from chronic hepatitis B accounted for about 820,000 deaths globally [1]. Timely diagnosis and treatment are essential to prevent these complications. This review focuses on the development of the new hepatitis B and D therapy that targets the various replicative sites of HBV and HDV.

## 2. HBV Structure and Life Cycle

**Figure 1:**
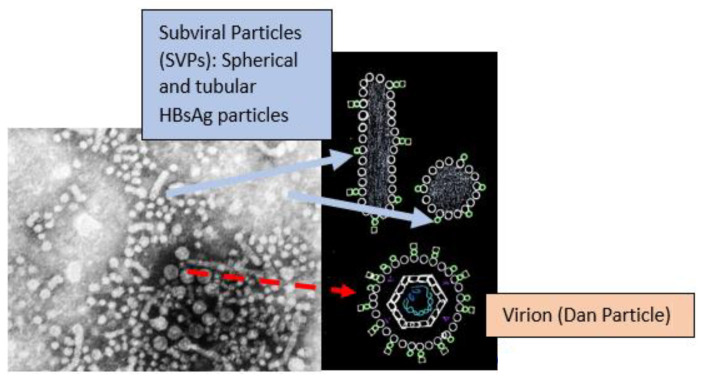
Electron microscope image has been taken from CDC’s public domain, and it is free of copyrights.

There are HBV genotypes from A to J with approximately 8% nucleotide variation in their genomes [4]. Genotyping is important as some are more likely to lead to chronicity [5] and affect the treatment course [6]. HBV consists of spherical and tubular (filamentous) subviral particles (SVPs) and replicative virions or Dane particles (Figure 1) [7].

The SVPs are non-infectious particles that contain only surface antigens (HBsAg) [8] which play a role in diverging the host immune response away from the virions [9]. SVPs are by far the most abundant source of HBsAg (>99.99% in the circulation) and are produced independently from viral replication and independently from cccDNA activity via integrated HBV DNA. HBsAg has important immunoinhibitory properties and its clearance during therapy is essential for achieving a functional cure [10]. Dane particles contain HBeAg, capsid (HBcAg), relaxed circular double-stranded DNA (rcDNA), DNA polymerase and reverse transcriptase [11]. The quantitative HBsAg test cannot distinguish HBsAg from the SVPs and virions in the serum [12].

**Figure 2:**
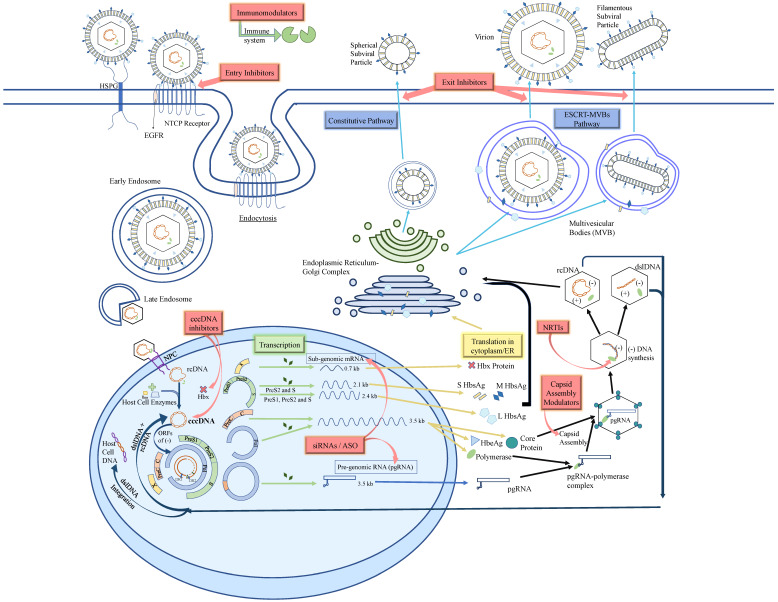
HBV life cycle in a hepatocyte. Entry inhibitors target the NTCP receptor on hepatocytes and hence prevent the entry of virion into the cell altogether. cccDNA inhibitors either act by targeting the HBx protein which leads to smc5/6 degrading cccDNA or by using the CRISPR/Cas9 nucleases system to break apart the cccDNA. Silencing RNAs (siRNAs) play a role in the destruction of newly formed RNAs. Antisense oligonucleotides (ASO) work against the mRNAs and stop them from reaching the translation step. Capsid assembly modulators cause the production of defected or empty nucleocapsids which fail to further produce new cccDNA or virions. Nucleic acid polymers (NAPs) block the assembly and the secretion of spherical SVP. The release of subviral filaments and virions is not affected [12]. Immunomodulators boost the immune system to attack the virus. HSPG: Heparan Sulfate Proteoglycans, NTCP: Sodium Taurocholate Cotransporting Polypeptide, EGFR: Epidermal Growth Factor Receptor, NPS: Nuclear Pore Complex, rcDNA: relaxed circular DNA, cccDNA: covalently closed circular DNA, ORFs: Open Reading Frames, dslDNA: double-stranded linear DNA, siRNAs: small interfering RNA, ASO: Anti-sense Oligonucleotide, S, M, L HBsAg: Small, Medium, Large HBsAg, ER: Endoplasmic Reticulum, ESCRT-MVBs pathway: Endosomal Sorting Complex Required for Transport.

To initiate the HBV life cycle (Figure 2), the newly formed HBV enters the bloodstream and attaches to the heparan sulfate proteoglycans (HSPGs) present on the cell surface membranes and gains entry into the hepatocyte via the sodium-taurocholate co-transporting polypeptide (NTCP) receptor [13] which is also used for the transportation of conjugated bile acids. The Large(L) HBsAg on the viral envelope forms an irreversible bond with the NTCP receptor and its co-receptor epidermal growth factor receptor (EGFR) [14]. The whole complex is then internalized into the cell via clathrin-mediated endocytosis or caveolin-dependent endocytosis [15].

Upon entering the hepatocyte, the endosomal shell carries the viral nucleocapsid containing relaxed circular DNA (rcDNA) across the cytoplasm to the nucleus [14]. The viral capsid attaches to the nuclear pore complex (NPC) and it is speculated that either the genome is released into the nucleus, or the entire capsid is internalized. This process can evade host-innate immunity cascade in the cytoplasm [16]. In the nucleus, the host cell enzymes DNA polymerases κ and α, DNA ligases 1 and 3 and topoisomerase I and II are hijacked by the virus to help complete the rcDNA into a complete covalently closed circular DNA (cccDNA). This is done by eliminating pol linked terminal redundant sequence on 5′end of negative (−) strand and RNA oligonucleotide(primer) on 5′end of positive (+) strand followed by the union of these two strands using ligases [17,18]. The (−) strand of rcDNA has four main open reading frames (ORFs); namely, the S, P, PreC + C and X ORFs [19]. Using the host cell machinery, the cccDNA is transcribed into subgenomic and pregenomic RNA (pgRNA) which move out into the cytoplasm. The mRNAs of the virus are translated into the large (L), middle (M), and small (S) surface protein (HBsAg), HBeAg, core (HbcAg), polymerase (pol) and HBx protein with regulation from enhancers and promotors [20]. pgRNA uses its cis-acting encapsidation signal known as epsilon (ε) to specifically bind with the newly translated polymerase in the cytoplasm [21]. The pgRNA-polymerase complex is then joined by dimers of capsid with the help of many cellular chaperones such as Hsc70, Hsp90 and Hsp40 [17,22]. This leads to the construction of a T-3 symmetry icosahedral nucleocapsid consisting of 30 nm 90 dimers or T-4 symmetry icosahedral nucleocapsid consisting of 34 nm 120 dimers around the pgRNA-polymerase complex [23]. Polymerase produces a 3-nucleotide RNA primer and proceeds to make (−) stand of Viral DNA using pgRNA as the template. After producing the negative strand (−), the RNAse H domain of polymerase disintegrates the pgRNA to make space for the (+) strand of DNA [17]. The making of (+) strand of rcDNA depends on the translocation of RNA primer from direct repeating 1 (DR1) to direct repeating 2 (DR2). If the primer remains bound to DR1, then the polymerase will proceed to produce dslDNA, the source of the DNA that can integrate into the host chromosome [24]. About 90% of the time, pgRNA in the nucleocapsids is reversely transcribed to produce rcDNA. A proportion of the rcDNA may be directed back to the nucleus to replenish the cccDNA. The majority of the rcDNAs are used to produce new virion particles in a process discussed further below [25]. The HBsAgs are produced using the ribosomes on endoplasmic reticulum (ER) from the mRNAs and are transported through the ER-Golgi-intermediate-complex (ERGIC) while being processed and packaged [26]. The spherical SVPs are secreted via the constitutive pathway, a pathway that allows for the continuous secretion of proteins without external signals [27]. The virions, on the other hand, undergo complex steps to form the multivesicular bodies (MVBs) and become enveloped with a lipid membrane embedded with HBsAg on its surface. They are subsequently secreted via the endosomal sorting complex pathway requiring a host cell protein called α-taxilin that coordinates intracellular vesicle trafficking [26]. The tubular (filamentous) subviral particles (SVPs) are secreted from the MVB pathway as virions [28,29].

## 3. Current Therapy of Chronic Hepatitis B 

Effective HBV vaccines have been available since the 1980s which led to a significant reduction in infection rates, particularly among children [30]. The effect of vaccines in preventing new infections is not hampered by their efficacy but by inadequate access to vaccines particularly in the resource-limited regions where the birth dose of the HBV vaccine is not uniformly provided [1]. Since vaccination does not help those with established infection, the prevalence of chronic hepatitis B has shown no tremendous decrease [31]. Interferon-α was the first approved medication for chronic hepatitis B. Both standard interferon-α (IFN-α), and later in 2005, the long-acting Pegylated interferon-α (Peg IFN-α) have both immunomodulatory and antiviral effects [32]. In 1998, lamivudine, the first nucleoside reverse transcriptase inhibitors (NRTIs) obtained Food and Drug Administration (FDA) approval. The NRTIs inhibit the reverse transcriptase enzyme competitively [33]. Over the years, other NRTIs were developed but lamivudine, adefovir and telbivudine are no longer considered first-line therapies due to the drug-associated resistance with their prolonged used [34]. Currently, entecavir, tenofovir disoproxil fumarate (TDF) and tenofovir alafenamide (TAF) are considered the first-line agents. Entecavir, however, should not be used for patients who have been on a prolonged course of lamivudine since it has a cross-resistance profile with lamivudine [35]. These NRTIs can optimally suppress HBV DNA in serum, normalize serum aminotransferases, prevent liver disease progression and reduce long-term complications such as hepatocellular carcinoma and liver failure. The loss of HBsAg or a functional cure with prolonged therapy over 5 years, however, occurs in less than 10% of patients [36]. NRTIs do not inhibit cccDNA directly and cannot eradicate cccDNA. A multi-center international study reported 9.1% HBsAg loss with a combination of Peg IFN-α + TDF compared to 2.8% with PEG-IFN monotherapy (*p* = 0.003) at week 72 after a 48-week treatment course [37,38]. Patients with HBV genotype A, lower HBV DNA level and elevated ALT levels tend to respond better to interferon-based therapy [38]. INF therapy is associated with significant adverse events and is a contraindication in patients with advanced liver disease. Due to these limitations with IFN, it is not considered as a first-line treatment for CHB [39]. Since relapse of HBV DNA in serum with potential hepatitis flares upon stopping therapy can be expected, treatment guidelines such as those from the American Association for the Study of Liver Diseases (AASLD) need to be followed, especially for patients with advanced hepatic fibrosis [40].

## 4. Definition of HBV Cure

The cure of HBV infection can be defined as a partial, functional, complete and sterilizing cure [41] (Table 1). HBeAg is nonreactive in all definitions of cure. Partial cure is achieved after a finite course of treatment when there is sustained low or undetectable HBV DNA level but HBsAg remains reactive; that is like an inactive carrier state. Functional cure, the current goal of therapy, is defined as loss of HBsAg in addition to sustained undetectable HBV DNA and normal ALT for 6 months after cessation of therapy. Complete or sterilizing cure is achieved with the addition of eliminating cccDNA and integrated HBV DNA from the hepatocytes. Complete cure is considered challenging because cccDNA persists even in patients with spontaneous recovery from acute hepatitis B [41]. Since functional cure is achievable in only 3–11% of patients after a prolonged course of current therapies [36,42], the immediate aim of the new HBV therapies is to achieve functional cure in a larger proportion of patients with a defined duration of treatment.

**Figure 3:**
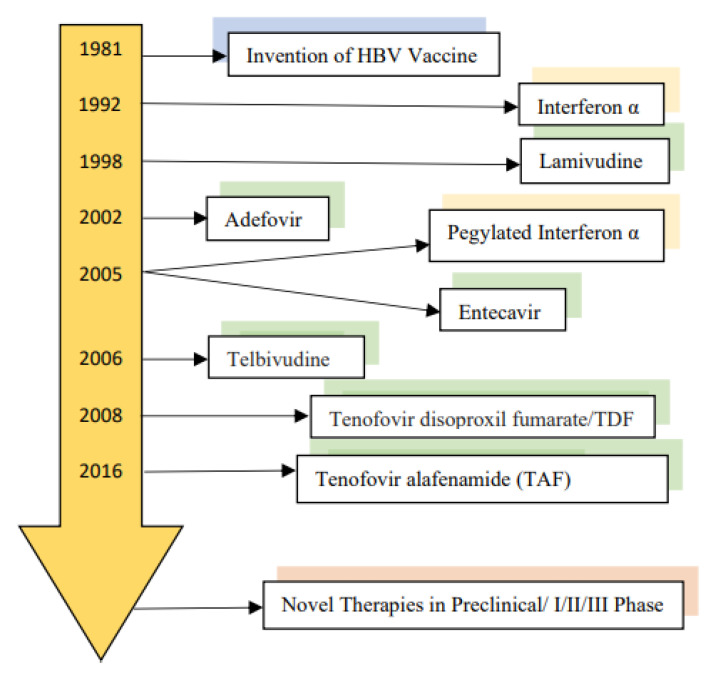
Development and approval of the HBV Therapy.

Many novel therapies with different mechanisms of actions are under preclinical, phase 1 and phase 2 clinical trials (Figure 3). These novel therapies include entry inhibitors, cccDNA inhibitors, silencing RNAs (siRNAs), antisense oligonucleotides (ASO), capsid assembly modulators, nucleic acid polymers (NAPs) and immunomodulators.

## 5. HBV Entry Inhibitor

Bulvertide (Myrcludex B) is a lipopeptide consisting of 47 amino acids corresponding to the pre-S1 domain of HBsAg that binds to sodium taurocholate co-transporting polypeptide (NTCP) of hepatocytes. NTCP links with HBV and transports it into the hepatocytes. Bile acids are increased due to this mechanism of action [43]. Bulvertide was first tested by Blank et al. in healthy volunteers, and it showed excellent tolerability [44]. Subsequently encouraging results were observed among patients with chronic hepatitis D. The results will be presented in the HDV therapy section.

## 6. cccDNA Inhibitors

The direct-acting cccDNA inhibitors can be classified as endonucleases. Zinc finger nucleases (ZFN), transcription-activator-like-effector nucleases (TALENs) and clustered regularly interspaced short palindromic repeats (CRISPR/Cas9) are three DNA cleaving enzymatic modules that can be used for making double-stranded breaks (DSBs). cccDNA, a covalently closed circular double-stranded DNA, serves as an ideal target. These DSBs are repaired using the error-prone non-homologous end-joining (NHEJ) pathway or the error-free homologous recombination (HR) pathway. The NHEJ pathway may produce a nonfunctional cccDNA with insertions, deletions, in frame or frameshift mutations [45]. All these compounds are in the early stage of development.

ZFNs were first designed with Zinc finger domains and Fok1 nuclease domain. The zinc finger domains recognize the specific site on DNA using its motifs and bind to it. The nuclease then trims off the DNA. However, the production of ZFN requires extensive protein engineering and a new ZFN needs to be created for every new genome target. It is not a clinically attractive therapeutic agent due to its high cost and time-consuming process [45,46].

TALENS used the repeat region of transcriptional activator-like effector (TALE) protein as the binding domain and Fok1nuclease for its endonuclease’s activities. It is technically demanding to engineer and require a specific TALEN for each DNA target site. There are also delivery issues as it is too large to be packaged using the Adeno-Associated Virus (AAV) vectors to the desired cells [45,47].

### 6.1. CRISPR/Cas9

CRISPR/Cas9 is being extensively evaluated in the preclinical phase. It cleaves DNA with its nuclease Cas9 at any site it is directed against using the synthetic single-guide RNA (sgRNA) [48]. This module is more cost-effective; it requires standard cloning techniques and is capable of targeting multiple areas of the DNA at the same time [45]. It has higher efficacy compared to ZFNs and TALENS. However, its large size makes it challenging to deliver using the AAV vectors [47].

In a recent CRISPR/Cas9 study, Stone, D. et al. used a liver-humanized FRG mouse model which ensured humanoid conditions for complete HBV replication cycle including the production of cccDNA in the mouse’s liver. They used a hepatotropic AAVLK03 vector to transport Staphylococcus aureus (Sa)Cas9 and 2 HBV-specific sgRNAs (C7 and C14) to chronically infected liver cells of eight mice. After the initial entecavir treatment, the mice were administered with anti-HBV AAV-SaCas9 vector or control anti-GFP AAV-SaCas9 vector. In this proof-of-concept experiment, treated mice but not controls showed a reduction of cccDNA levels. This study also reported an increased survival of hepatocytes with anti-HBV AAV-SaCas9 treatment compared to the control group. Clinical validation of the model is necessary for HBV DNA levels, and cccDNA copies per cell are usually much higher in patients [49].

### 6.2. HBx Inhibitors

The host cell’s structural maintenance of chromosomes 5/6 complex (Smc5/6) aims to degrade the cccDNA in the nucleus. The HBx protein intervenes by manipulating the host cell’s DNA damage binding protein 1 (DDB1) E3 ubiquitin ligase to degrade the Smc5/6, hence keeping cccDNA intact. A study was conducted using HBV minicircle DNA as a surrogate to cccDNA to evaluate the effects of Nitazoxanide (NTZ). NTZ successfully inhibits this HBx-DDB1 protein interaction and substantially restores Smc5/6 levels. This causes the suppression of viral transcription and viral protein production [50]. The DDB1 E3 ubiquitin ligase process requires the neuronal precursor cell-expressed developmentally down-regulated protein 8 (NEDD8) for activation. Pevonedistat, a NEDD8-activating enzyme inhibitor, indirectly inhibited DDB1 protein and restored Smc5/6 levels that, in turn, led to the reduction of HBV DNA transcription and protein production [51].

## 7. Small Interfering RNA (siRNA)

RNA interference (RNAi) is a post-transcriptional gene silencing mechanism that is affected by small duplex RNAs such as the 21–23 nt short interfering RNAs (siRNAs) and the ≈22 nt microRNAs (miRNAs) [52]. miRNAs are transcribed from specific genes as precursor transcripts which undergo modification by the nuclear Drosha-DGCR8 microprocessor complex and RNase III enzyme Dicer to produce ≈ 22 bp miRNA duplex. This is an imperfect duplex having 2 nt 3′ overhangs [46]. One of the two miRNA strands is selected as mature or guide to be loaded onto the Argonaute (AGO) protein of the RNA-induced silencing complex (RISC) [53]. RISC then binds to the complementary biding sites in the 3′ untranslated region of the messenger RNAs leading to their deadenylation, translational repression or degradation. If there is near-perfect complementary base pairing between miRNA and mRNA, the Guide RNA-AGO complex degrades the target mRNAs [54].

Since synthetic siRNAs induce RNAi, these can be used to suppress the replication of hepatitis B by targeting HBV RNA. HBV produces overlapping transcripts with a common 3′ end [55,56], a single siRNA; therefore, it could simultaneously affect many viral mRNAs. In order to deliver siRNAs to the hepatocytes, vectors are needed because naked siRNAs cannot enter the cells. Both non-viral vectors such as lipid nanoparticles, stable nucleic acid particles, N-acetylgalactosamine (GalNac/NAG) as well viral vectors have been evaluated. GalNac conjugated siRNAs have become a popular choice for liver-targeted delivery [57]. Similarly, viral vectors, especially adeno-associated viral vectors, have demonstrated good efficacy [58].

Antisense oligonucleotides (ASO) interfere with HBV protein expression by degrading the viral mRNAs. These single-stranded fragments of DNA or RNA bind to the complementary sequence of mRNAs through base pairing to form hybrids of DNA: RNA (antisense DNA) and duplexes of RNA: RNA (antisense RNA), respectively. Subsequently, the host Ribonuclease H-dependent mechanism degrades the mRNAs transcripts and silences the protein expression [59,60].

### 7.1. ARC 520

ARC 520 is conjugated to cholesterol for enhanced delivery to hepatocytes. It is coinjected with liver-targeted Dynamic PolyConjugates (DPC). DPC is a polymer-based system that uses melittin-like peptide that helps endosomal escape and cytoplasmic delivery of siRNA to hepatocytes [61].

In an early study on chimpanzees, the animals were initially treated with daily oral NUCs for 8–24 weeks, then in combination with ARC-520 injections every 4 weeks for a total of 24–48 weeks. A 4.0 ± 0.2 log10 copies/mL reduction in DNA levels was seen with NUCs in HBeAg(+) chimps. An additional decrease of 1.3 ± 0.1 log10 in HBV DNA occurred with the first injection of ARC-520. Interestingly, the decrease in HBsAg was significantly more pronounced in HBeAg(+) compared to HBeAg(−) chimps. The failure for ARC-520 to reduce HBsAg among HBeAg(−) subjects was due to the loss of ARC-520 target sites in the mRNA encoded by the integrated DNA. Subsequently, a second-generation siRNA, siHBV-75, was developed that included target sites upstream from the DR1-DR2 region. With this siRNA modification, the reduction in HBsAg was similar in both HBeAg (+) and (−) chimpanzees [62].

The other part of this study was conducted in humans when nucleotide analogue (NUC) treated patients were given the improved siRNA. A sharp decrease in HBsAg was observed during the first eight days with a maximum reduction of 0.3 log10 seen in patients with a maximum dose of 4 mg/kg. Unlike the first-generation siRNA, HBeAg status did not appear to affect the overall reduction in HBsAg. A mean reduction of 0.9 log10 HBcrAg was seen in both HBeAg (+) and HBeAg (−) NUC-treated cohorts receiving 4 mg/kg dose. Among the treatment naïve patients, the reductions of HBsAg (1.4 ± 0.1 log10), HBeAg (1.5 ± 0.1 log10) and HBcrAg (1.3 ± 0.1 log10) were greater than the NUC-experienced patients [62]. An extension of the above study for 3 HBeAg (+) and 5 HBeAg (−) patients in the treatment naïve cohort who received 13 doses of ARC-520 (4 mg/kg) with NUCs for 28.9–30.4 months led to a further reduction in all the HBV parameters including HBV RNA, HBcrAg, and HBsAg in both HBeAg (+) and HBeAg (−) patients. HBsAg seroconversion occurred in one HBeAg (+) and one HBeAg (−) patient. HBeAg seroconversion was observed in two out of three HBeAg (+) subjects [63].

The ARC-520 study emphasized the importance of including target sites in the mRNA encoded by the integrated DNA. Arrowhead, however, stopped the development of ARC-520 due to the deaths of nonhuman primates. That was likely due to liver toxicity from the EX1 dynamic DPC delivery vehicle, a version of GalNAc-conjugated melittin-like peptide [64].

### 7.2. JN-3989 (ARO-HBV)

This compound is designed to silence all mRNAs from both the cccDNA and integrated DNA. It is composed of two siRNAs, each conjugated to a triantennary N-acetyl galactosamine cluster (GalNAc) for effective hepatic delivery. A recent phase II study reported safety, tolerability and pharmacokinetics in both healthy individuals and CHB patients. After three monthly doses of ARO-HBV, a reduction in HBV DNA, RNA, HBeAg, HBsAg and HBcrAg was evident in CHB patients. Mean NADIR HBsAg reduction in patients with >6 weeks of HBsAg data was 2.1 log10 in HBeAg (+) and 1.8 log10 in HBeAg (−) patients. While > 1 log reduction of HBsAg occurred in 100% of the patients, only 3% had >3 log reduction. No patient dropouts or serious adverse effects were reported [65]. Preliminary results of a phase 2 study were presented at the American Association for the Study of Liver Diseases (AASLD) meeting in 2019. A total of 48 treatment-naïve or NUC-experienced CHB patients were given three subcutaneous doses of 25, 50, 100, 200, 300 or 400 mg at days 1, 27 and 57. At day 113, ≥1 log10 reduction in HBsAg was seen in 100% of the patients treated with 200, 300 and 400 mg; 87.5% (7/8) and 50% (4/8) achieved ≥ 1 log10 reduction with 100 and 25 mg, respectively. Reduction in HBV DNA, HBV RNA, HBeAg and HBcrAg was also observed. There were no serious drug-related adverse events or treatment discontinuation noted [66]. With longer follow up of the 100–400 mg dose groups, sustained reduction of HBsAg (defined as ≥1 log10 reduction about 9 months after the last dose) was noted in 56% (22/39) and occurred more frequently in patients who had greater reductions of HBsAg levels. Sustained suppression was also observed for HBV RNA, HBeAg and HBcrAg in (58%) 15/26, (62%) 9/14 and (42%) 10/24 of the patients respectively [67].

### 7.3. AB-729

This RANi is covalently conjugated to triantennary GalNAc. In an early phase study, healthy individuals and non-cirrhotic CHB patients with undetectable HBV DNA and ALT ≤ 5 X ULN were given a single dose of various concentrations of AB-729. At 12 weeks, a mean HBsAg reduction of 0.99 log10, 1.31 log10 and 0.98 log10 was noted with the 60, 90 and 180 mg dose, respectively. No serious adverse events or discontinuation due to adverse events were noted [68].

### 7.4. VIR-2218 

VIR-2218 is a GalNac conjugated RNAi that inhibits mRNA from both cccDNA and integrated HBV DNA. VIR-2218 was created using Enhanced Stabilization Chemistry Plus to reduce the off-target effects by enhancing in vivo metabolic stability. It showed favorable pharmacokinetics in healthy individuals after subcutaneous dosing [63,69]. The interim results of a phase 2 trial were presented at the European Association of the Study of the Liver (EASL) liver congress in 2020. A total of 24 noncirrhotic patients with HBeAg (+) or HBeAg (−) CHB who had suppressed HBV DNA on NUCs were enrolled. Patients were administered two subcutaneous doses of VIR-2218 (20–200 mg) or placebo on day 1 and day 29. A rapid early decline in serum HBsAg was observed with all the doses within 8 weeks. A maximum decline of 1.5 log10 from baseline was observed in a subset of the patients treated with the 50 mg dose and a decline of 1.0 log10 was maintained through 28 weeks of follow up [70]. The final data after a follow up of 32 weeks of this phase 2 clinical trial were presented at the EASL in 2021. Most participants achieved a maximum decline at week 16 of treatment. In HBeAg (+) patients, a maximum decline of 1.16 log10 and 1.57 log10 was noted with doses of 50 mg and 200 mg. The maximum reduction in HBsAg in HBeAg (−) patients were 1.03 log10, 1.23 log10, 1.50 log10 and 1.65 log10 at doses of 20, 50, 100, 200 mg, respectively. Similarly, HBeAg and HBcrAg levels also decreased in HBeAg (+) patients. No serious adverse events or significant ALT elevations were observed [71]. The efficacy of VIR-2218 is also being evaluated in combination with peg-IFN. Preliminary data from a phase II study after 12 weeks show a steeper and more profound decline in HBsAg in combination therapy as compared to VIR-2218 alone. A total of 27 participants of the study were treated with 200 mg of VIR-2218 subcutaneously every 4 weeks for 6 doses either alone (cohort 1), or in combination with 12 weeks of 180 mcg peg-IFN-α starting on week 12 of VIR-2218 treatment (cohort 2), or in combination with 24 weeks of 180 mcg peg-IFN-α concurrently (cohort 3). A total of 14 patients completed 12 weeks of treatment at the time of presentation; the mean reduction in HBsAg for the 10 patients in cohorts 1 and 2 was 1.0 and 1.1 log10 IU/mL, respectively. A total of four patients in cohort three had a mean HBsAg reduction of 1.8 log10 IU/mL. Some patients experienced asymptomatic ALT flares with concurrent HBsAg decline in the combination therapy arm. No serious adverse effects were reported [72]. 

### 7.5. RG-6346

RG-6346 is a GalNac conjugated dsRNAi which induces the cleavage of all the mRNA copies from both cccDNA and integrated DNA. The safety and efficacy of RG-6346 were assessed in healthy individuals and non-cirrhotic CHB patents with HBsAg > 1000 IU/mL in HBeAg(+) or HBsAg >500 IU/mL in HBeAg(−) ones. There were three groups: Group A consisted of 30 healthy individuals in which 20 were given 0.1, 1.5, 3.0, 6.0 and 12.0 mg/kg of RG-6346 and 10 were treated with placebo. Group B consisted of nine treatment-naïve CHB patients with HBV DNA > 2000 IU/mL, ALT ≥ ULN. Six of nine patients were given a single 3.0 mg/kg RG-6346 dose and then NUCs were added after 12 weeks. Group C consisted of 18 NUC-experienced CHB patients with suppressed HBV DNA. A total of 12 patients in Group C were given 4 monthly doses of 1.5 (*n* = 4), 3.0 (*n* = 4) and 6.0 (*n* = 4) mg/kg. A total of six were placebo-treated. In group B, the mean HBsAg reduction from baseline was 0.69 log with HBV DNA decreased from 7.20 IU/mL at baseline to 5.98 IU/mL on day 85 of therapy. In Group C, mean HBsAg reduction of 1.39, 1.80 and 1.83 log10 IU/mL on day 112 was observed with 1.5, 3 and 6 mg/kg dosing, respectively. A total of 82% of the patients who reached at least day 112 achieved > 1.5 log10 IU/mL reduction and 64% achieved HBsAg < 100 IU/mL, regardless of the HBeAg status. No serious adverse effects, dose-limiting toxicities or withdrawals from treatment were observed [73].

## 8. Anti-Sense Oligonucleotides (ASO)

### 8.1. GSK-3389404

GSK-3389404 is a triantennary GalNAc conjugated ASO that targets RNAs and degrades them via the RNase H-dependent pathway. In a phase I study, it was found to be safe both in healthy individuals and CHB patients. Mostly mild or moderated side effects such as upper respiratory tract infections, headache and injection site reactions were noted. Two subjects had a transient grade 1 increase in hepatic enzymes [74]. In a phase 2a multicenter, randomized, double-blind and placebo-controlled study, 66 CHB patients with suppressed HBV DNA were given either GSK-3389404 60 mg weekly, 120 mg weekly, 120 mg biweekly or placebo for 12 weeks. At day 85, a maximum reduction of 0.75 log IU/mL in HBsAg was observed in patients taking the 120 mg weekly dose. Only three patients, one from each GSK-3389404 dosing group, had ≥ 1.5 log reduction in HBsAg at day 85. Two patients, one each in 120 mg weekly and 120 mg bi-weekly dose group, had hepatitis flares with peak ALT at 182 U/L and 113 U/L; the flares were associated with a decrease in HBsAg titers. An early dose-dependent decrease in platelet count was also observed. It plateaued on treatment and recovered after treatment completion. No bleeding events were reported [75]. The development of this compound was later halted due to low efficacy.

### 8.2. GSK-3228836 (ISIS-505358)

GSK-3228836 is an ASO that inhibits all mRNA copies. It shows good pharmacokinetics and antiviral effects in a recent phase II study. Both HBeAg(+) and HBeAg(−) CHB patients who were either on NUC therapy for ≥12 months with suppressed DNA (<20 IU/mL) or treatment-naïve with DNA ≥ 2000 IU/mL were enrolled. ASO 300 mg or placebo were given subcutaneously on days 1, 4, 8, 11, 15 and 22. On day 29, the NUC-experienced patients had 2.5 log reduction in HBsAg with ASO therapy vs. 0.008 log with placebo. For the treatment-naïve patients, a 1.556 log reduction of HBsAg was observed. Most of the treatment-naïve and NUC-treated patients had acceptable tolerability and safety in this study. Injection site reactions were the most common side effects observed. However, significant hepatitis flare with ALT peaked at 781 U/L and 15X ULN after ASO therapy was observed in two subjects [76]. The mechanisms of the hepatitis flare are being carefully evaluated.

### 8.3. RO-7062931 (LNA-SSO) 

RO-7062931 is a GalNac conjugated ASO with locked nucleic acid and causes RNase H mediated degradation of all the mRNAs. A total of 59 CHB patients with HBsAg ≥ 1000 IU/mL, HBV DNA ≤ 90 IU/mL and ALT ≤ 1.5 × ULN were randomized either to receive RO-7062931 or placebo. Over the course of four weeks, six treatment regimens consisting of 2–5 subcutaneous injections of 0.5, 1.5, 3 or 4 mg/kg were given according to the required dosing frequency i.e., monthly, bi-weekly or weekly. The dose-dependent decrease in HBsAg levels was noted regardless of the HBeAg status. However, a rebound in HBsAg levels was observed 2–3 weeks after treatment and returned to baseline levels by week 12. No serious adverse effects or withdrawals were reported. Only one patient had a transient ALT elevation of >3xULN that was associated with declining HBsAg levels [77]. Despite the early promise, the development of the compound was cancelled due to poor efficacy.

In all clinical studies with RNAi and ASO monotherapy to date, HBsAg decline was minimal or suboptimal in 40–50% of the patients. In the phase II study, Peg-IFN in addition to VIR-2218 showed a much more profound decline in HBsAg compared to VIR-2218 alone. This provided clues that an immunomodulator is likely needed to achieve functional cure in combination with RNAi or ASO.

## 9. Core Protein Allosteric Modulators (CpAMs)

The core protein (Cp) is composed of 183 fragments and forms the basic framework of the nucleocapsid. The *N*-terminal region mediates the assembly function and maintains the homodimer structure of the Cp. A total of 120 core dimers ultimately multimerize to form the icosahedral nucleocapsid. The Cp has multifaceted functions such as housing/encasing HBV genome. Its arginine abundant *C*-terminal domain, in particular, mediates pgRNA capsidation, synchronizes reverse transcription and causes intra-nuclear transport by enabling the entry via the nuclear pores [78].

In general, CpAMs interfere with the assembly process primarily by covalently attaching to the hydrophobic pockets at the dimer–dimer interface, leading to covalent modifications in the quaternary structure of the protein [79]. In addition, some CpAMs cause indirect inhibition of cccDNA by disintegrating nucleocapsid uncoating during the first few steps of the HBV viral cycle [80].

### 9.1. Types of CpAMs

HBV capsid assembly is a highly organized process. Two different classes of CpAMs are identified which can affect this process. Class I CpAMs or Heteroaryldihydropyrimidines (HAPs) cause the desynchronized assembly of the capsids, forming aberrant or abnormal non-capsid polymers which cause the disintegration of the core proteins [81]. Class II CpAMs are Phenylpropenamides and Sulfamoylbenzamides which speed up the assembly process to an extent that the HBV pgRNA does not get inserted inside the capsid, resulting in an empty capsid. Many drugs under Phase 1 or 2 clinical developments are Class II CpAMS [78].

#### 9.1.1. Class 1 CpAM

##### R07049389

In Phase I multi-center, placebo-controlled, randomized clinical trial, patients with CHB who were not currently on therapy were randomly allocated to different multiple ascending dose cohorts for 28 days. It was found that regardless of the doses allocated (200 mg or 400 mg twice a day, or 200 mg, 600 mg, or 1000 mg once a day), the mean HBV DNA decline was more than 2.5 IU/mL and HBV RNA decrement followed the same pattern. When the treatment was discontinued, HBV DNA and RNA levels returned to the baseline. No apparent changes, however, were observed in the HBeAg or HBsAg levels after 28 days of dosing with RO7049389 or placebo. The most common adverse events noted were a headache and elevation of ALT and AST, which returned to baseline during the follow-up period. This drug has progressed to longer-term phase II clinical [82,83].

#### 9.1.2. Class 2 CpAMs

##### ABI-HO731

In a multi-center, Phase-I, randomized, placebo-controlled study conducted for 28 days by Yeun M-F et al., it was found that ABI-HO731 caused dose-dependent reductions in HBV DNA and RNA. Mean maximum HBV DNA declines from baseline were 1.7 log, 2.1 log and 2.8 log in the 100 mg, 200 mg and 300 mg dose cohort, respectively. There was no change in HBsAg levels. No serious adverse effects were noted, and the pharmacokinetics supported daily dosing for ABI-HO731 due to its long half-life and rapid absorption [84].

ABI-HO731 progressed to phase II drug trial which assessed the antiviral effects with the combined use of nucleos(t)ide analogues (NA) and 300 mg daily ABI-HO731 for 24 weeks. The combination therapy exhibited greater reductions in HBV DNA compared to NA with a 5.94 log decrease in HBV DNA and 2.54 logs decline in pgRNA. Combination treatment for 48 weeks resulted in further reductions in HBV DNA (6.3 log) and pgRNA (3 log). There was an associated decrement in HBcAg (1.42 log), HBeAg (1.03 log) and HBsAg (0.86 log) levels [85].

In another combination drug trial by Gane et al., viral response and safety were assessed after discontinuing ABI-H0731 and NRTIs combination therapy. The first part of the study 201 was double-blinded. Virologically-suppressed cHBV patients received ABI-H0731 +NRTI or placebo+NRTI for 24 weeks. Patients who completed study 201 were further enrolled in the open-label ABI-H0731 (300 mg once daily) +NRTI Study 211 up to 76 weeks. Treatment was discontinued in patients who met the stopping criteria: HBV DNA < 20 IU/mL and HBeAg negative OR ≤ 5 IU/mL for ≥6 months prior to treatment Week 76. Following the discontinuation of ABI-H0731 +NRTI, a post-hoc analysis was performed. All the patients relapsed off-treatment by week 16. None of the patients achieved sustained virological responses and had HBV DNA > 20 IU/mL by post-treatment week 16. None of the patients experienced HbsAg loss [86].

##### NVR-3778

NVR-3778 is the first Class II CpAM that became available in the oral drug form. In a 4-week, phase 1, multi-center placebo-controlled trial, NVR3-778 exhibited a minimal decrease in HBV DNA. Combination therapy of NVR3-778 (600 mg twice daily dose) and pegylated-IFN showed a synergistic decrease in HBV DNA with a mean reduction of 1.97 log. After the discontinuation of the therapy, HBV DNA and RNA levels returned to baseline levels. There was no significant reduction in HBsAg, HBcAg or HBeAg levels. No serious side effects were recorded [87].

##### ABI-H2158

ABI-H2158 is a second generation CpAM inhibitor. In a phase 1b multi-center multiple ascending dose cohort, placebo-controlled study on HBeAg (+), treatment-naïve CHB patients for 14 days, Kosh Agarwal et al. observed dose-dependent reductions in HBV DNA and pgRNA with 100, 300 and 500 mg daily dose. With the 300 mg dose, there was a 2.5 log and 2.3 log reduction in HBV DNA and pgRNA, respectively. There was no change in HBsAg, HBcAg or HBeAg titers [88]. In a recent multicenter, randomized, placebo-controlled drug trial conducted in California, the effects of ABI-H2158 in 88 treatment-naïve patients were assessed. They were stratified based on HBeAg positive or HBeAg negative CHB without cirrhosis. Patients were randomly allocated 3:1 to receive either ABIH2158 or placebo, in addition to entecavir, for 72 weeks. Severe ALT grade 4 surge was found in two patients and a grade 3 ALT elevation was found in two patients. As no other cause could be elucidated, it was concluded that ABI-H2158 resulted in hepatotoxicity which led to the discontinuation of the drug for further development [89].

##### JNJ-6379

JNJ-6379 was evaluated in a phase 1b, randomized, double-blind, placebo-controlled study for 4 weeks in treatment-naïve, non-cirrhotic CHB patients. It exhibited a safe clinical profile with no detrimental drug-related side effects. There was a significant decline of 2.16–2.89 log in HBV DNA and 1.67–2.3 log in RNA levels. No significant changes in HBsAg titer were observed. JNJ-6379 has progressed to phase II clinical trials [90]. In the phase 2b REEF-1 study, 57.5% of patients achieved HBsAg < 100 IU/mL on a combination of JNJ-6379 and JNJ-3989 (siRNA), which was lower than the 74.7% on JNJ-3989 alone [91]. Despite the early promise, the development of this compound was halted due to poor efficacy.

##### JNJ-0440

In a phase Ib randomized placebo-controlled drug trial with multiple ascending dose cohorts, mean HBV DNA reduction at 28 days was 3.2 log with the 750 mg daily group and 3.3 log with the 750 mg twice-daily group. There was no decrease in HBsAg levels. There was, however, a 0.2 log decline in mean HBeAg with both dosing regimens. In addition, HBcrAg decreased by 0.62 logs and 0.85 log with the once-daily dose of 750 mg and twice-daily dose of 750 mg, respectively. With these promising results, this compound has progressed to phase II trial [92].

For all CPAMs assessed to date in clinical studies, they do not have direct inhibition to cccDNA. The reduction of HBcrAg and HBeAg is absent or mild, relative to NUCs [93,94,95]. Newer generations of CPAMs with more potent effects on reducing cccDNA are in development.

## 10. Nucleic Acid Polymers (NAPS)

Nucleic acid polymers (NAPs) are oligonucleotide, amphipathic and water-soluble compounds. The phosphothioration of the non-bridging oxygen atom in the phosphodiester linkage enhances the amphipathic properties. NAPs interact with the amphipathic alpha-helical domains in multiple infectious agents, including HBV and hinder their entry and replication in the host cells [96,97]. NAPs also block the assembly and release of subviral particles into the circulation system. The surface glycoprotein of many viruses has an analogous amphipathic protein that makes these drugs have a very broad spectrum in targeting viruses [97,98,99]. NAPs have no effect on cccDNA transcription, HBV RNA translation or the production and secretion of HBeAg or Dane particles in HBV infection. Boulon R et al. reported that DNAJB12 is a HSP40 chaperone required for the assembly of spherical subviral particles at the 2020 AASLD meeting [100]. These investigators presented updated data at the 2021 HBV International meeting confirming DNAJB12 is targeted by NAPs, and the antiviral interaction is ph-dependent [101].

In the open-label REP 101 study by Al-Mahtab et al. [102], eight treatment-naïve CHB patients from Bangladesh were treated with NAP, REP 2055 and monotherapy. A total of 7/8 patients experienced 2.03–7.2 log reduction in HBsAg as compared to baseline. Serum HBeAg and HBV DNA levels were also reduced in 7/8 and 6/8 patients, respectively. Asymptomatic serum transaminase elevations were reported in four patients immediately after declining HBsAg levels and self-resolved without any complications. At 23 weeks, the drug was well tolerated with manageable adverse effects. Only grade 1–2 adverse effects were reported and responded well to acetaminophen or antihistamine. Treatment with 1200 mg REP 2055 was stopped in only one patient due to mild bleeding and elevated INR.

In REP 102 study by Al-Mahtab et al., 12 Bangladeshi patients were treated with REP 2139-Ca monotherapy, and 9/12 were transitioned to combination therapy with pegINF-α or thymosin alpha 1 immunotherapy. In total, 9/12 patients experienced 2.79–7.10 log reduction in HBsAg during monotherapy and later achieved HBsAg loss with combination therapy. Loss of HBeAg was achieved in 8/9 HBeAg(+) CHB patients. Asymptomatic serum transaminase elevations were reported in five patients related to HBsAg reduction. Adverse events were reported mostly with combination therapy that included low-grade dyspepsia, fever, moderate dysphagia, hair loss and reduced appetite [102].

Bazinet et al. reported the results of the Phase 2 clinical trials on REP 2139-Mg or REP 2165-Mg [103]. Initially, all the patients were treated with tenofovir disoproxil fumarate (TDF) for 24 weeks. Subsequently, 20 patients in the experimental arm received TDF, pegylated interferon alfa-2a (pegIFN) and REP 2139-Mg or REP 2165-Mg for 48 weeks, and 20 patients in the control arm received TDF and pegIFN for 24 weeks followed by 48 weeks of TDF, pegIFN and REP 2139-Mg or REP 2165-Mg combination therapy. All the patients were observed for 48 weeks off therapy. In the experimental group, HBsAg levels significantly declined in 19/20 patients and 4–6 log_10_ reductions were reported in 15/20 patients. Seroconversion was reported in 11/20 patients by the end of 48 weeks. In the control group, >1 log_10_ IU/mL reduction in HBsAg was only noted in 3 of 20 patients during TDF and pegIFN therapy. After 48 weeks with the addition of NAP, 12 of 20 had seroconversion. There was no significant difference in efficacy between REP 2139-Mg or REP 2165-Mg. During the 48 weeks of treatment-free follow-up, functional cure with HBsAg seroconversion persisted in 14 of 40 participants. Hepatitis flares were significantly high in the NAPs group. The elevation in transaminases correlated with the decrease in HBsAg levels and were not associated with worsening of the liver function tests. Viral rebound with hepatic decompensation was reported in one patient. The high rate of functional cure associated with NAPs is very encouraging. Long term efficacy and safety monitoring are important.

## 11. Novel Immunomodulators

Activation of the immune system is essential in achieving HBV functional cure. Patients with controlled HBV infection generally have activated humoral and cellular immunity producing HBV specific antibodies, cytotoxic and helper T cells [104].

Unlike acute HBV infection with spontaneous recovery, patients with CHB are noted to have dysfunctional and exhausted HBV specific CD4+ and CD8+ T cells, dysfunctional and reduced number of dendritic cells (DCs) and natural killer/natural killer T cells (NKs/NKTs). In addition, there are elevated regulatory factors such as immune check point proteins PD-1, CTLA4, T cell immunoglobulin domain and mucin domain along with down regulation of toll-like receptors, which are critical innate immune system factors [105]. Novel therapies that aim to activate the immune system may prove vital in achieving functional cure [106].

### 11.1. TLR 7 and TLR 8 Agonists

Toll like receptors (TLRs) are genetically encoded pattern recognition receptors found on the surface of cells. TLRs recognize pathogen-associated molecular proteins (PAMPs) [107,108]. These compounds (TLR7, TLR8 agonists) play a crucial role in activating an innate immune response in the liver as well as linking it with an adaptive immune response via adhesion to neighboring immune cells of hepatocytes. A newly developed TLR7 agonist (GS-9620) successfully induced IFN-alpha production from plasmacytoid dendritic cells (pDCs) in the gut and liver after oral administration [106]. The administration of this drug in HBV infected woodchucks and chimpanzees demonstrated rapid and sustained anti-HBV activity which makes it a potential candidate for achieving functional cure [109].

When tested in phase I/II clinical trial, GS-9620 had no adverse effects but lacked significant antiviral activity probably because of the decreased dose used in patients (4 mg/patient contrast to 1 mg/kg in chimpanzees) [110]. Novel TLR8 agonists show a strong affinity for PAMPs and produce strong IFN-gamma responses in the liver due to their ability to induce IL-12 and IL-18 production from monocytes and DCs of the liver. These cytokines induce NK and NKT cells, specifically mucosa-associated invariant T (MAIT) cells in the liver, to produce a large quantity of IFN-gamma. Moreover, the ability of IL-12 to enhance the partial recovery of exhausted HBV specific T cells provides evidence that it also boosts HBV specific T cell immunity, in addition, to direct antiviral effects by inducing IFN-gamma production [111].

### 11.2. RIG-I Activator

SB9200 activates RIG-I and nucleotide-binding oligomerization domain-containing protein 2 (NOD2), and preferentially induces interferon-mediated pathways in hepatocytes and blood. When administered in HBV infected Woodchucks, SB9200 produced a dose-dependent decline in HBV DNA and HBsAg levels accompanied by the correlated presence of IFN-α or IFN-β [112]. In human trials, it produced a dose-dependent decline between 0.58 and 1.54 log HBV DNA as well as a reduction in RNA levels, especially among HBeAg negative patients. In addition, HBsAg levels decreased by 0.5 log at 24 weeks of therapy in 33% (16/49) of responders [104]. The phase 2b trial was prematurely terminated after the occurrence of unexpected serious adverse events, including one death.

### 11.3. PD-1/PDL-1, CTLA4 Check Point Inhibitors

PD-1 is a cell surface receptor present on activated B and T cells. In CHB, the interaction of PD-1 with its ligands on the hepatocyte limits T-cell activation and inhibits the downstream antiviral signaling pathways via T-cell receptors [113,114]. It has been demonstrated that PD-1 is overexpressed in HBV specific T cells in chronic infection. The blocking of PD-1 and its ligands by the check point inhibitors may lead to the revival of exhausted T-cells and restore its antiviral function. Check point inhibitors are novel therapies for CHB. Its efficacy has been demonstrated in both animal models and in in vitro studies [115,116].

In transgenic mice models, HBV specific T-cells rapidly produced IFN-gamma upon exposure to the hepatic HBV antigens. The production of IFN-gamma was suppressed with increased PD-1 expression on CTLs. With anti-PD-1 antibodies treatment, there was a delay in the suppression of IFN-gamma production and importantly, a proliferation of IFN-gamma producing T-cells was observed [117].

Besides the upregulation of PD-1 on T-cells, upregulation of PDL-1 on hepatocytes in CHB have been described and could contribute to T-cell exhaustion. PDL-1, however, was also increased in non-viral hepatitis and other inflammatory conditions making it difficult to predict whether PDL-1 is associated with T-cell exhaustion, or if it is a counteractive homeostatic mechanism to control inflammation [114,118]. Moreover, T-cells exhaustion is not solely a result of the overexpression of PD-1/PDL-1; other inhibitors may act synergistically leading to T-cells dysfunction. Combination therapy to target these interactive pathways will likely be necessary to restore the T-cell functions [119,120].

ASC22 (Envafolimab) is a subcutaneously administered monoclonal antibody against PD-L1. Results of a Phase IIa clinical trial was presented at the 2021 AASLD meeting by Wang G and coworkers. It was a single-dose escalation study of three subcutaneously administered doses (0.3, 1.0 and 2.5 mg/kg, three patients per dose) with a 12-week follow-up to explore the safety and preliminary efficacy of ASC22 in CHB patients that were all on nucleoside analogs treatments. There was a trend of dose-dependent HBsAg reduction from 0.08 to 1.2 log IU/mL after single-dose administration. ASC22 was well tolerated with only grade 1 adverse effects reported [121].

Similar to PD-1, CTLA-4 (CD-152) is expressed on the surface of T-cells. It inhibits the activation of T cells by either directly inhibiting the TCR signaling or by blocking B7, a target for CD28. During chronic HBV infection, CTLA-4 expression is increased on T-cells along with other inhibitory molecules which contribute to the exhaustion of T-cells [122,123].

## 12. Engineered HBV Specific T-Cells

Recently, attempts have been made to devise a therapy that specifically targets HBV infected hepatocytes. Genetically engineered T cells have been made which either carried a chimeric T-cell receptor (cTCR) containing anti-HBs-specific antibody or HBV specific T-cell receptor gene transferred through a vector. In in vitro studies and in animal models, these engineered T-cells (eTC) successfully recognized HBV infected cells exhibiting HBV proteins on their surfaces. This resulted in the elimination of HBV infected cells and a substantial decrease in cccDNA. These promising observations support the development of eTC in a clinical setting though it is technically challenging [106,124,125,126].

## 13. Therapeutic Vaccines

**Figure 4:**
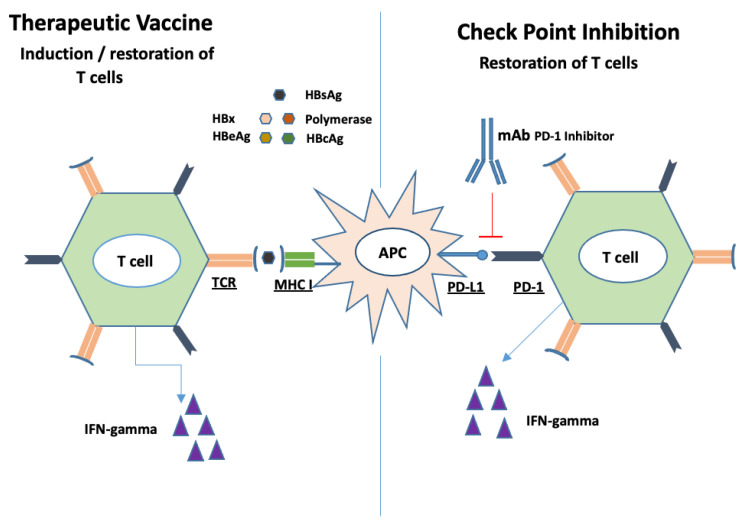
Restoration and induction of HBV specific T cells. Therapeutic vaccines consist of various HBV antigens which are processed by APCs. These APCs present the antigens either to new T cells inducing their function or exhausted T cells restoring their function (**Left**). Check point inhibitors block the interaction of PD-1 with PD-L1 thus boosting T cells function (**Right**).

Unlike preventive vaccines, therapeutic vaccines are administered to treat instead of preventing infections. The major mechanism of action is the induction of CD4 and CD8 T cell responses. HBV vaccines interact with professional antigen-presenting cells (APCs) such as DCs. These APCs then induce HBV specific T cells to release antiviral cytokines such as IFN-gamma, as shown in Figure 4 [127].

Evidence from HBV mice model reported favorable efficacy with therapeutic vaccines. TG1050 is an immune therapeutic vaccine composed of three HBV antigens; namely, HBsAg, HBcAg and polymerase. When injected to HBV infected mice, TG1050 induced HBV specific T cells which caused a significant reduction in HBV DNA and HBsAg. These antiviral effects were long-lasting up to 1-year post-HBV injection. Buchmann et al. used HBsAg and HbcAg in combination with saponin based ISCOMATRIX adjuvant as a therapeutic vaccine and treated the transgenic (HBVtg) mice. The vaccine was able to induce HBsAg and HBcAg specific CD8+ T cells in the spleen, and HBcAg specific CD8+ T cells in the liver along with HBsAg seroconversion 2 weeks post-injection. It also reduced HBcAg expression in the liver of the HBVtg mice [128,129]. To date, the therapeutic vaccines tested in humans have not been able to replicate these encouraging results in animal models [129,130,131,132]. The failure of these vaccines can be partially attributed to their inability to induce HBV-specific T cells in the setting of high-level circulating HBsAg. Recently, other HBV antigens such as HBcAg and polymerase have been evaluated as potential therapeutic vaccines.

As monotherapy, the therapeutic vaccines have not been effective in restoring the exhausted T cells. When combined with check point inhibitors such as PD-1 blockers, increased efficacy of therapeutic vaccines was observed in the LCMV mouse model and HBV infected woodchucks. DC-induced HBV-specific T cells were more responsive to PD-1 blockers compared to hepatocyte induced T cells [133,134,135]. Yet these promising results, again, could not be replicated in the clinical setting. In a small pilot study, 10 HBV DNA suppressed patients were treated with a combination of GS-4774 (a therapeutic vaccine) and 0.3 mg/kg nivolumab (PD-1 blocker) and 14 patients with nivolumab alone (0.3 mg/kg *n* = 12, 0.1 mg/kg *n* = 2). No change in HBsAg from baseline was observed with nivolumab 0.1 mg/kg, but there was a significant reduction in HBsAg from baseline with nivolumab 0.3 mg/kg and nivolumab 0.3 mg/kg + GS-4774. There was, however, no significant difference in HBsAg reduction between the nivolumab 0.3 mg/kg and nivolumab 0.3 mg/kg + GS4774 at weeks 12 (*p* = 0.24) and week 24 (*p* = 0.39) post-treatment. The reason for lack of significance could be due to the lack of immunogenicity of the vaccine or the timing of the PD-1 blocker administration [136].

## 14. Novel HDV Therapies

Hepatitis D virus (HDV) has a rod-like genome consisting of approximately 1700 nucleotides and is the smallest single-stranded RNA virus that can infect humans. NTCP receptor is the gateway for HDV and HBV into hepatocytes [137]. The HBsAg envelope of HDAg consists of about 95% P24/GP27s, 5% GP33/36s and 1% P39/GP42S proteins. This protein composition is similar to that of the 22-nm particles of HBsAg. This provided evidence that the HDAg production and HDV maturation proceed in patients without the complete HBV virions [138]. The high GC content of its genome causes increased intramolecular base pairing that renders rod-like confirmation to it. This structure resembles double-stranded DNA, and the host RNA polymerases get tricked, so they continue to copy the viral RNA as if it were endogenous. This is pivotal for HDV to complete its replicative cycle as the antigen itself has no RNA polymerase to replicate its genome [139,140]. As a result, direct inhibition of viral replication with polymerase inhibitors, therefore, is not possible. Treatment strategies are aimed at tackling the entry, immune modulation and secretion of viral particles. Until recently, the only recommended therapy for HDV therapy was pegylated interferon-α (PEG IFN-α) for 48 weeks; however, this has limited efficacy and poor tolerability [141].

### 14.1. PEG IFN-α

The Hep-Net–International Delta Hepatitis Intervention Trial (HIDIT-1) [142] treated 90 chronic hepatitis D (CHD) patients with PEG IFN-α 2a for 48 weeks, with or without Adefovir. The sustained HDV RNA clearance was reported in about 25% and sustained biochemical response in 40% of patients. In the follow-up HIDIT-II [143], 120 patients were randomly assigned to receive either PEG IFN-α 2a (180 μg once weekly) plus TDF (300 mg daily) or PEG IFN-α -2a (180 μg once weekly) plus placebo for 96 weeks. Increasing the duration of PEG IFN-α 2a therapy to 96 weeks was possible in most patients, with an acceptable, expected safety profile and it resulted in achieving HDV suppression in about 40% of patients achieving virological response. The addition of Adefovir or TDF did not result in improvement in the rate of HDV clearance in HIDIT-I and HIDIT-II. Heller. T et al. [144] reported that extending PEG IFN therapy beyond 1 year or increasing the dose to 270 mg/week did not improve the rates of complete virologic response.

### 14.2. PEG IFN-LAMBDA (PEG IFN-λ) 

PEG IFN-λ is a novel type III interferon and the IFN-λ receptor (IFNLR1) is preferentially expressed in epithelial tissues of the lung, liver and gut [145]. It has less of the typical IFN alfa related side effects. A phase 2 randomized clinical trial of 33 CHD [146] patients treated with Peg IFN-λ 180µg monotherapy for 48 weeks demonstrated 36% durable virologic response 24 weeks post-treatment compared to PEG IFN-α (28%). It was well tolerated by the majority of the patients. Patients who were previously treated with interferon Alfa noted significantly fewer side effects on Peg IFN-λ. Cases of jaundice and bilirubin elevation were reported. There was no incidence of hepatic decompensation and bilirubin normalized with dose reduction or dose discontinuation. Peg IFN-λ is currently being evaluated in combination with Lonafarnib and Ritonavir in a phase II trial (LIFTstudy) [147]. In this trial, 96% of the patients had a >2 log HDV RNA decline and 58% achieved below the limit of quantification (BLQ) or undetectable HDV RNA after 24 weeks of treatment.

### 14.3. Lonafarnib (LNF)

Lonafarnib is a tricyclic, non-peptidic farnesyl-transferase inhibitor that was developed originally in 1998 for anticancer therapies [148]. It was found to prevent the release of HDV particles in 2003. A crucial step in the assembly of HDV virion particles are prenylation, that is, the covalent addition of a prenyl lipid group to the HDV large antigen (HDLAg) post-translation and is accomplished by enzyme Prenyl transferase (Farnesyl transferase) [149].

In a phase 2A double-blind, randomized, placebo-controlled study [150] of LNF for 28 days, there was a dose-dependent decline in HDV RNA at end of therapy from baseline. A 0.13 log (95% CI −0.14 to 0.34) with placebo, 0.73 log (0.17–1.31) with LNF 100 mg twice daily and 1.54 log (1.21–1.93) with LNF 200 mg twice daily (BID) were reported. No serious adverse events were reported. However, gastroenterology side effects such as nausea, diarrhea, bloating/dyspepsia and decreased appetite were noted with the higher dose. It was subsequently found in the Lonafarnib with Ritonavir for HDV-1 (LOWR HDV-1) study [151] that the addition of Ritonavir (RTV), a cytochrome P450 3A4 inhibitor, increased the bioavailability of LNF. Thereby, a lower dose of LNF can be used to reduce the GI side effects.

The LOWR HDV-2 study [152] was aimed at identifying the appropriate combination regimens of LNF and RTV ± PEG IFN-α. Safety, tolerability and efficacy were examined. The addition of PEG IFN-α 180 mcg weekly to LNF 25 mg BID + RTV 100 mg resulted in the highest response with HDV RNA reduced to less than the limit of quantitation (LOQ) at Week 24 in all five patients. That demonstrated the synergistic effects of the LNF and PEG-IFNα combination.

LOWR HDV-3 [153] was aimed to assess the antiviral effects and safety and tolerability of once-daily RTV boosted LNF. A total of 21 patients with CHD were enrolled in a double-blinded, randomized, placebo-controlled study receiving either LNF 50/75/100 mg + RTV 100 mg once daily for 24 weeks (12 patients) or 12 weeks of placebo followed by LNF 50/75/100 mg + RTV 100 mg once daily for 12 weeks (9 patients). HDV RNA levels significantly declined during the 24 weeks of LNF 50/75/100 mg + RTV 100 mg once daily and 6/21 (29%) patients achieved HDV viral RNA levels < 250 IU/mL. ALT normalized in 66% of the patients. Adverse events were mild to moderate, which included nausea, vomiting, dyspepsia, anorexia, diarrhea and weight loss.

The primary objective of LOWR HDV-4 study [154] was to evaluate the strategy of dose-escalation and maintenance of LFN with RTV for 24 weeks. A total of 15 patients were enrolled. All 15 patients were treated with LFN 50 mg BID plus ritonavir 100 mg BID initially. LFN was increased to 75 mg BID after 4 weeks and subsequently to 100 mg BID after additional weeks. The dose was escalated in 10/15 (66%) of patients but only 5 were able to tolerate LNF 100 mg BID + Ritonavir 100 mg BID for 24 weeks. At the end of 24 weeks of therapy all 13/15 patients who completed therapy, achieved HDV RNA decline with 9 patients (60%) had >1 log decline while 4 (27%) had >2 log decline. HDV RNA was undetectable in one patient. A total of 53% of the patients had normalized ALT at week 24. Gastrointestinal adverse events were mostly from grade 1 to 2.

Koh C et al. [155] evaluated the safety and efficacy of LNF boosted with RTV and PEG IFN- λ in a phase IIA open-label study. A total of 26 CHD patients were treated with LNF 50 mg BID, RTV 100 mg BID and PEG IFN-λ 180 mcg weekly for 24 weeks with 24-week post-treatment follow up. TDF or ETV started to suppress HBV prior to the therapy. A total of 19 out of 26 subjects reached the end of therapy. The median HDV RNA decline was 3.4 log and seven patients (37%) achieved undetectable HDV RNA. Adverse events were mostly mild to moderate with weight loss, hyperbilirubinaemia and anaemia. Therapy was dose reduced in three patients and discontinued in four patients.

LNF has been granted Orphan Drug Designation as well as Fast Track Designation and Breakthrough Therapy Designation by the FDA.

### 14.4. Entry Inhibitor

Myrcludex B (MyrB), now known as Bulevirtide (BLV, Hepcludex^®^), was approved in 2020 in the European Union for adult patients with CHD and compensated liver disease at 2 mg daily subcutaneous administration [156]. FDA approval is still pending.

In a multicenter, open-label phase 2 trial (MYR202), 120 patients were treated with either tenofovir alone or Myr B (at 2, 5, or 10 mg) in combination with tenofovir for 24 weeks to assess its safety and efficacy. At the end of therapy, a dose-dependent decline in HDV RNA between 1.6 log and 2.7 log in the Myr B-treated arm was seen. There was, however, no reduction in HBsAg levels. At 12 weeks of follow-up, 80% of the patients who responded to Myr B had a relapse of HDV RNA [157].

The final results of another phase 2 trial that was designed to evaluate the efficacy of Myr B and PEG IFN-α 2a combination therapy were recently published by Wedemeyer, H et al. A total of Sixty HBeAg-negative CHD patients were divided into four groups and received: 180 mg PEG IFN-α alone, Myr B alone, Myr B 2 mg with PEG IFN-α or Myr B 5 mg with PEG IFN-α for 48 weeks followed by a follow-up period of 24 weeks. Combination therapy with PEG IFN-α plus Myr B 2 mg per day showed promising results with a decrease in HDV RNA of 4.81 log at the end of the therapy which was maintained at 4.04 log reduction at 24 weeks post-treatment. With this regimen, 50% of the subjects achieved undetectable HDV RNA, 40% with 1 log reduction in HBsAg levels and 47% had ALT normalization at 24-week follow up [158]. This combination regimen was well tolerated with some expected side effects relating to PEG IFN-α. The main adverse events from Myr B were related to the elevation of bile acids. Thus, careful monitoring of total bile acid levels during therapy is essential [158].

Current data suggest that Myr B can be used as a suppressive strategy alone or as a curative strategy in combination with PEG IFN in selective patients. The optimal duration, long-term safety and higher doses of Myr B are currently being investigated in the MYR301 trial. Data on the safety of patients with decompensated cirrhosis are currently lacking.

### 14.5. Nucleic Acid Polymers (NAPS)

Given the promising results with HBV infection alone, REP 301 and REF 301-LTF clinical trials on HBV and HDV co-infected patients were conducted [159]. Twelve CHD patients received weekly intravenous (IV) REP 2139-Ca (500 mg) for 15 weeks followed by 15 weeks of IV REP 2139-Ca (250 mg) combined with PEG IFN-α and finally, PEG IFN-α alone for 33 weeks. A total of 11 out of 12 (91%) patients became HDV RNA negative during treatment, with 9 (75%) remaining HDV RNA negative at the end of treatment, and 7 (77%) had sustained undetectable HDV RNA by the end of 1-year follow-up. The most common reported side effects during treatment were thrombocytopenia, neutropenia and increased ALT levels.

To further assess the long-term safety, tolerability and persistency of virological control and functional cure, 11 participants were followed for 3.5 years in the REP 301-LTF study. Sustained virological response persisted in 7 of 11 (63%) participants with no additional safety or tolerability issues observed [160]. These results look very promising. More extensive studies are critical to understanding the mechanisms of action and safety profiles of these compounds.

## 15. Summary/Future Prospects

Hepatitis B virus is a DNA virus affecting 296 million individuals globally and is a significant cause of mortality. The current therapy requires chronic suppression of the HBV and only about 10% of the treated patients can achieve functional cure. HDV is an RNA virus that coinfects or superinfects on HBV. There are many novel therapies for chronic hepatitis B and D in development. The goal of HBV therapy is functional cure with HBsAg clearance. Since HDV requires HBsAg to replicate, the functional cure for HBV can also lead to the eradication of HDV. Many novel therapies targeting different steps of the viral life cycle have shown promising results in phase 1 and 2 clinical trials against both viruses. With various promising direct antiviral compounds and immunomodulatory agents in development, it would be possible to evaluate combination therapies that hopefully can lead to a definite cure with a defined duration of therapy. The safety profiles of the medications, however, need to be carefully determined in parallel to the assessment of their efficacy. With focused efforts and determination, HBV and HDV could become the next viruses to be eliminated.

## Figures and Tables

**Table 1:** Definition of partial, functional and complete HBV cure.

Partial Cure	Functional Cure	Complete Cure
HBV DNA low or not detected	HBV DNA not detected	HBV DNA not detected
HBeAg negative	HBeAg negative	HBeAg negative
HBsAg positive	HBsAg negative ± anti-HBs	HBsAg negative ± anti-HBs
cccDNA detected	cccDNA detected but not active	cccDNA not detected
Integrated HBV DNA detected	Integrated HBV DNA detected	Integrated HBV DNA not detected

## Data Availability

Not Applicable.
